# Validation of a modified version of the adult developmental eye movement test

**DOI:** 10.1038/s41598-021-99245-5

**Published:** 2021-10-05

**Authors:** Andrés Gené-Sampedro, Pedro Miguel Lourenço Monteiro, Inmaculada Bueno-Gimeno, Javier Gene-Morales, David P. Piñero

**Affiliations:** 1 Department of Optics, and Optometry and Vision Science, University of Valencia, Valencia, Spain; 2 INTRAS (Research Institute on Traffic and Road Safety), University of Valencia, Valencia, Spain; 3 Department of Physics, University of Beira Interior, Covilhã, Portugal; 4 CICS (Health Sciences Research Centre), University of Beira Interior, Covilhã, Portugal; 5 (PHES) Prevention and Health in Exercise and Sport Research Group, University of Valencia, Valencia, Spain; 6 Department of Optics, Pharmacology and Anatomy, University of Alicante, Crta San Vicente del Raspeig s/n, 03690 San Vicente del Raspeig, Alicante Spain

**Keywords:** Eye manifestations, Oculomotor system

## Abstract

This study evaluates in terms of reliability, internal consistency, and validity a modification of the Adult Developmental Eye Movement (ADEM) test, ADEM with distractors (ADEMd), designed to analyse oculomotor system, visual processing and visual attentional behaviour. 302 healthy subjects participated in the study (20–86 years old). Intrasession repeatability was evaluated by analysing the correlation between the time needed to read different parts of the test. Inter-session analyses were carried in 40 subjects by calculating intraclass correlation coefficients and using the Bland–Altman method. Validity was assessed in the outcomes obtained according to age as well as investigating the correlation between ADEMd and attentional useful field of vision (UFOV) test. Correlation coefficients between times need to read each sheet were ≥ 0.95 (p < 0.001). The inter-session intraclass correlation coefficient ranged from 0.81 in the horizontal distractor sheet to 0.97 in the vertical sheet. Bland–Altman analysis showed clinically acceptable limits of agreement. Statistically significant correlations were found between age and ADEMd outcomes (r ≥ 0.55, p < 0.001). Processing velocity, divided attention and selective attention measured with the UFOV were correlated with the horizontal distractor times (r ≥ 0.32, p < 0.001). ADEMd test may be a useful clinical tool to evaluate the combined interaction of ocular movements and visual attentional behaviour.

## Introduction

The Developmental Eye Movement test (DEM) is an easy test developed in 1990 to easily characterize the ocular movements during reading in children^1^. It consists of series of simple numbers that are recognized and verbalized out loud allowing the clinician to measure the speed and precision of ocular movements while reading these numbers. Specifically, the relationship between the horizontal and vertical tasks allows identifying a tracking problem (saccades) and or a difficulty in the visual-verbal automaticity of verbalizing numbers^1^. The design of the test combines saccadic ocular movements, spatial tracking, and the ability to verbalizing^2^. Although there is an influence of the motor and cognitive components of the speech and language as well as of the visual attentional behaviour on the outcomes obtained with the DEM test, clinicians have found this test as an easy, practical, and economic mode of indirectly evaluating the ocular movements involved in reading processes^2^. Ayton et al.^3^ found that DEM outcomes cannot be directly correlated with specific parameters of ocular movements. However, these authors found that the test outcomes were related to reading development and speed of visual processing, with the potential of being a diagnostic test in clinical practice^3^. Other authors have also shown that reading ability is highly correlated with the speed of temporal processing^4,5^. Medland et al.^6^ suggested that the DEM test should not be used to diagnose eye movement difficulties in patients with poor reading ability. Concerning the reliability of the DEM test, it has been shown to be good^1,7^, especially in those children with symptoms related to an oculomotor dysfunction^7^.

The DEM test has been suggested to be potentially useful in adults, especially in screening saccadic eye movements after brain injury^8,9^. However, the guidelines for the interpretation of the data obtained with this test is based on the outcomes obtained in a children population and therefore it cannot be extrapolated to adults. For this reason, our research group developed in 2003 a DEM version for adults, which was designated as Adult Developmental Eye Movement Test (ADEM)^10^. The ADEM is similar to DEM but includes two-digit numbers in order to increase the difficulty to compensate for the increase in age^10^. This version was developed in a Spanish-speaking population with ages between 14 and 68 years and afterwards validated in English-speaking subjects^11^. This test has been used to evaluate differences between drivers and non-drivers with interesting results^12^.

Both mentioned tests (DEM and ADEM) require visual (central and peripheral) and cognitive (automaticity of number naming) attention and processing, as well as vertical and horizontal saccades of varying magnitude^10,11^. These tests involve (as in usual reading) the use of overt (with fixations) and covert (without fixations) attention to reading the numbers as fast as possible^13,14^. The DEM and ADEM are used for evaluating what Powell et al.^11^ called saccadic efficiency (i.e., indirect evaluation of saccadic function in combined tracking and cognitive visual-verbal identification), rather than directly measuring the eye movements. However, there is still some controversy on whether the DEM estimates the quality of saccades or only the reading performance^3,15^, with a study asserting that the DEM test could replace an eye-tracker examination^15^. In this sense, a recent study^16^ evaluated ocular movements in the DEM by means of an eye tracker. They encountered poorer vertical and horizontal eye movement control, longer fixations, and poorer test performance in below-average reading ability children and, therefore, this test could be useful in both research and clinical settings^31^.

The aim of the current study was to validate in terms of reliability, internal consistency, and validity a modification of the ADEM test (ADEMd). The modification consists of including an additional sheet of numbers combined with letters to increase the difficulty of the cognitive processing as explained further below.

## Results

### Intrasession repeatability

In the sample of healthy subjects, the Spearman correlation coefficient associated with the relationship between the adjusted time required to name the numbers of the first vertical sheet and that required for naming the number of the second vertical sheet was 0.98 (p < 0.001). Likewise, the correlation coefficient associated with the relationship between the adjusted time required to read the first 40 numbers of the horizontal sheet and that required for naming the other 40 numbers of the horizontal sheet was 0.95 (p < 0.001). The correlation coefficient associated with the relationship between the adjusted time required to read the first 40 numbers of horizontal distractor sheet and that required for naming the other 40 numbers of horizontal distractor sheet was 0.96 (p < 0.001).

### Test–retest analysis

**Figure 1:**
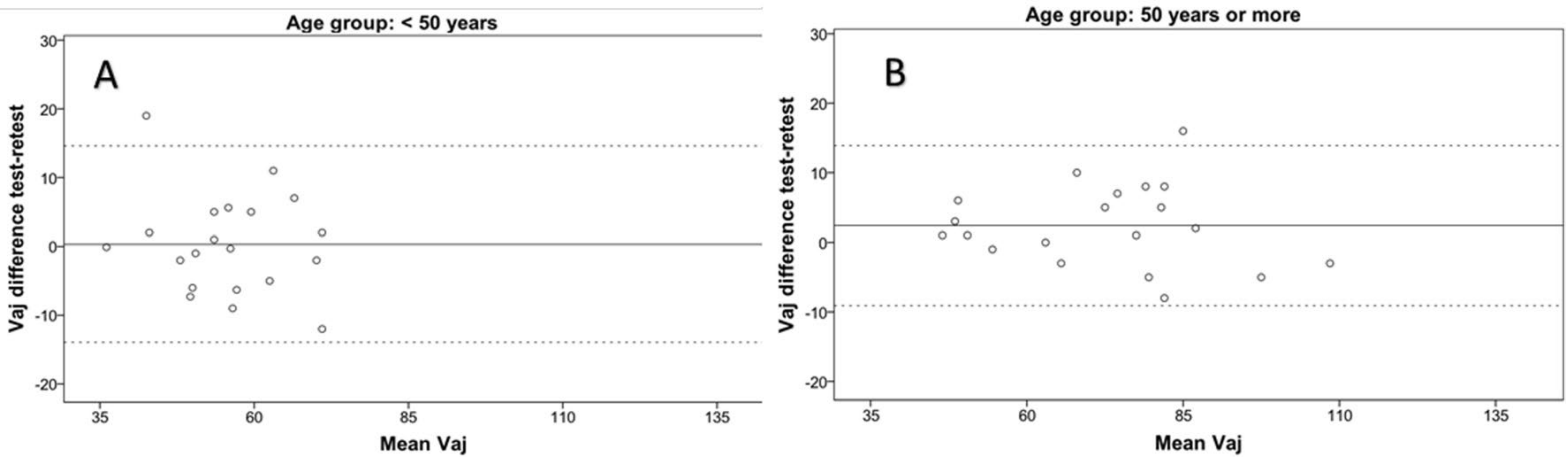
Test–retest Bland–Altman analysis of the vertical sheets in subjects with less than 50 years (**A**) and subjects with 50 years old or more (**B**). Data of both axes are presented in seconds. The continuous horizontal line represents the mean difference and the two non-continuous lines the limits of agreement. *Vaj* vertical adjusted time.

**Figure 2:**
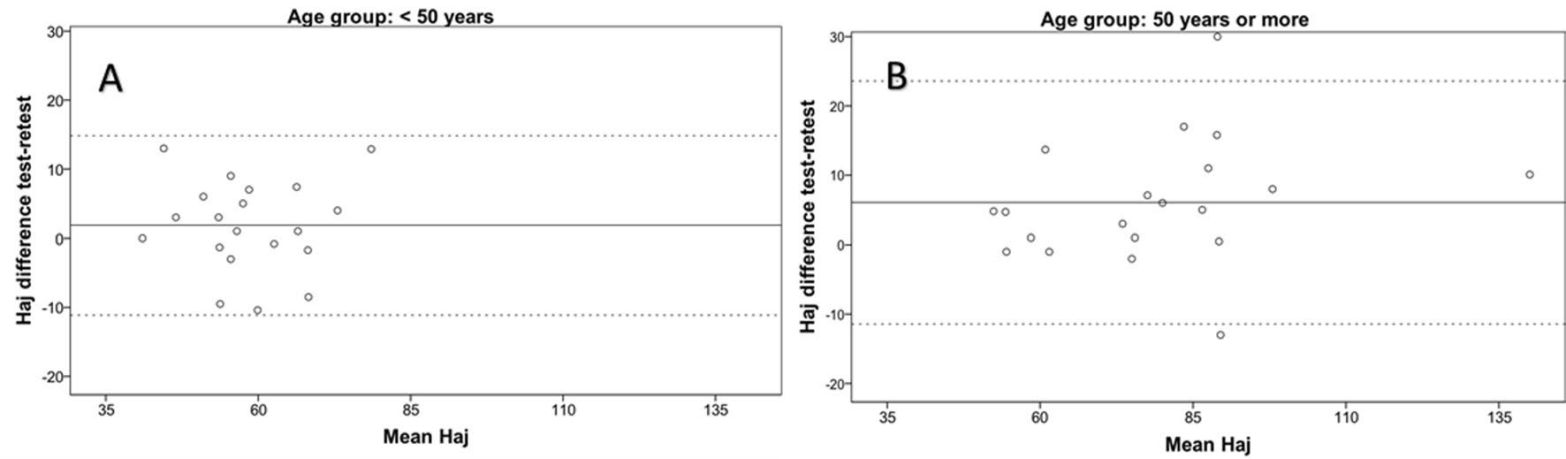
Test–retest Bland–Altman analysis of the horizontal sheet in subjects with less than 50 years (**A**) and subjects with 50 years old or more (**B**). Data of both axes are presented in seconds. The continuous horizontal line represents the mean difference and the two non-continuous lines the limits of agreement. *Haj* horizontal adjusted time.

**Figure 3:**
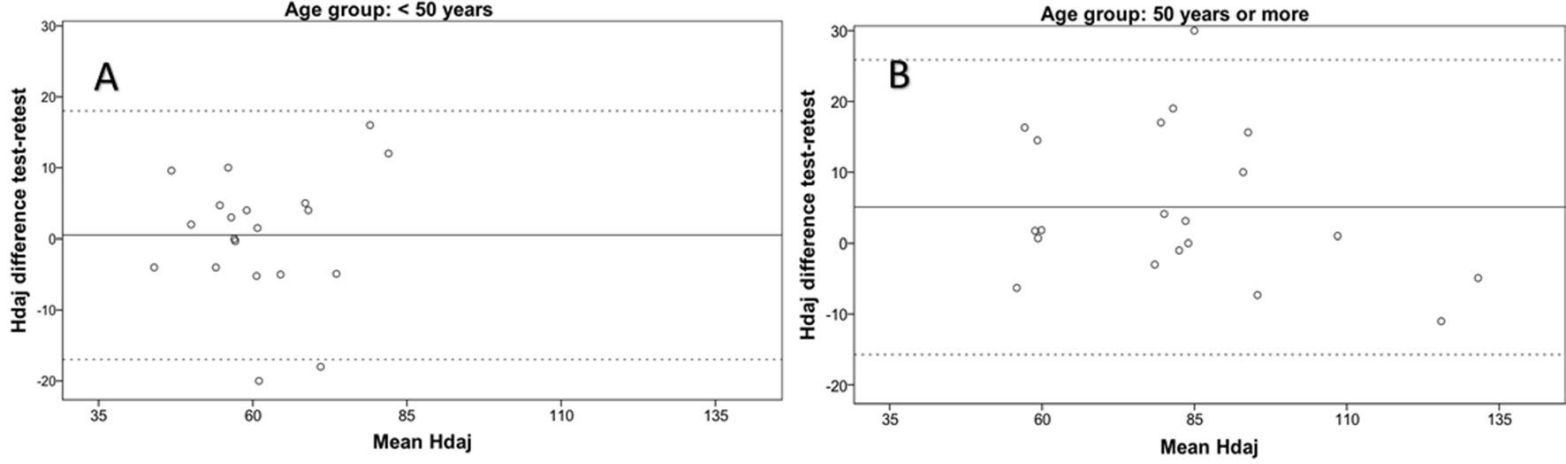
Test–retest Bland–Altman analysis of the horizontal distractor sheet in subjects with less than 50 years (**A**) and subjects with 50 years old or more (**B**). Data of both axes are presented in seconds. The continuous horizontal line represents the mean difference and the two non-continuous lines the limits of agreement. *Hdaj* horizontal distractor adjusted time.

Table 1 summarizes ICC results obtained in the test–retest analysis organized according to age. Figures 1, 2, and 3 display the outcomes of the Bland and Altman analysis in which clinically acceptable limits of agreement are displayed (below 35 s in all cases).

**Table 1:** Test–retest analysis of the adjusted times for the vertical and horizontal sheets of the ADEMd, the sample is subdivided according to age (younger than 50 years and 50 years old or more).

Age group		Test Median (IQR)	Retest Median (IQR)	ICC (CI 95%)
**Vaj (s)**				
< 50		55.0 (16.5)	54.8 (11.5)	0.87 (0.67–0.95)
≥ 50		77.5 (29.3)	73.0 (24.3)	0.97 (0.92–0.99)
**Haj (s)**				
< 50		58.5 (13.0)	56.5 (14.5)	0.88 (0.70–0.95)
≥ 50		82.1 (30.1)	75.0 (24.5)	0.93 (0.83–0.97)
**Hdaj (s)**				
< 50		59.5 (15.5)	58.7 (17.0)	0.81 (0.54–0.93)
≥ 50		84.6 (33.9)	79.0 (28.5)	0.93 (0.82–0.97)

*Vaj* vertical adjusted time, *Haj* horizontal adjusted time, *Hdaj* horizontal distractor adjusted time, *s* seconds, *IQR* interquartile range, *ICC* intraclass correlation coefficient, *CI* confidence interval.

### Validity analysis

Statistically significant differences were found in all temporal parameters measured with the ADEMd test in different age subgroups (all p < 0.001, Kruskal–Wallis test) (Table 2).

**Table 2:** Results of the ADEMd test in different age subgroups. The median and the interquartile range is displayed for each subgroup and each parameter as well as the p-value obtained of the global comparison among age subgroups.

Age groups	≤ 24 n = 26	25–34 n = 55	35–44 n = 31	45–54 n = 32	55–64 n = 65	65–74 n = 61	≥ 75 n = 32	p-value
V1 (s)	26.5 (6.0)	27.5 (8.5)	25.0 (9.0)	28.5 (7.8)	30.0 (13.0)	36.3 (17.1)	42.0 (19.8)	< 0.001
V1aj (s)	26.5 (6.0)	27.5 (8.5)	25.0 (9.0)	28.5 (7.1)	30.0 (13.0)	36.3 (17.1)	42.0 (19.1)	< 0.001
V2 (s)	25.5 (3.5)	26.2 (8.0)	25.0 (6.0)	27.5 (8.9)	28.0 (11.0)	35.0 (17.3)	40.0 (18.2)	< 0.001
V2aj (s)	25.5 (3.5)	26.2 (8.0)	25.0 (6.0)	27.2 (8.9)	28.0 (11.0)	35.0 (17.3)	40.0 (17.4)	< 0.001
Vaj (s)	52.0 (7.0)	54.3 (14.6)	49.0 (14.1)	55.5 (17.8)	57.7 (23.6)	73.0 (30.4)	82.0 (38.2)	< 0.001
H (s)	55.1 (7.5)	57.0 (13.5)	53.0 (14.0)	61.5 (18.5)	66.0 (22.0)	75.0 (32.5)	91.0 (38.3)	< 0.001
Haj (s)	55.5 (7.5)	57.0 (13.5)	54.0 (14.0)	61.5 (18.5)	65.0 (21.5)	69.1 (36.5)	92.1 (35.3)	< 0.001
Hd (s)	57.0 (9.3)	59.0 (10.5)	60.0 (16.0)	67.5 (20.8)	67.0 (21.5)	78.0 (29.5)	90.0 (27.3)	< 0.001
Hdaj (s)	56.9 (8.0)	59.0 (10.4)	60.0 (16.3)	67.5 (20.9)	67.9 (20.1)	80.0 (28.8)	90.5 (26.9)	< 0.001

^a^Data are presented as median (IQR), or p values where indicated.

*H* horizontal time, *Haj* horizontal adjusted time, *Hd* horizontal distractor time, *Hdaj* horizontal distractor adjusted time, *Vaj* vertical adjusted time, *V1* first vertical sheet time, *V1aj* first vertical sheet adjusted time, *V2* second vertical sheet time, *V2aj* second vertical sheet adjusted time.

**Figure 4:**
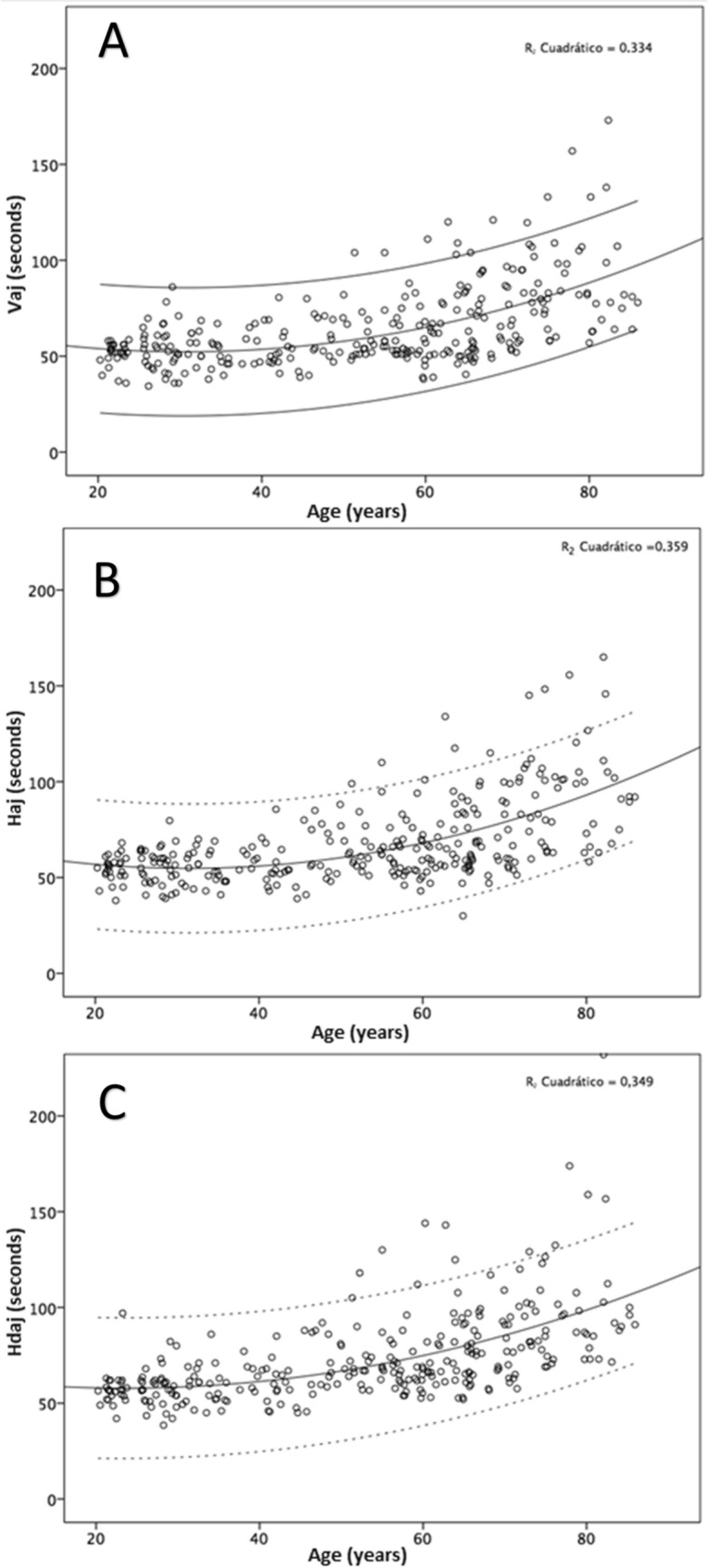
Scatterplot showing the correlation between the age of participants and the ADEMd outcomes: vertical adjusted time (**A**), horizontal adjusted time (**B**), and horizontal distractor adjusted time (**C**). A curvilinear trend, represented by the solid line, is observed in the results of all the sheets. Confidence intervals at 95% are pictured by the dotted lines. *Haj* horizontal adjusted time, *Hdaj* horizontal distractor adjusted time, *IC* confidence interval, *Vaj* vertical adjusted time.

A statistically significant positive correlation was found between age and the three adjusted times (Spearman correlation coefficient: vertical adjusted r = 0.55, Horizontal adjusted r = 0.55, and Horizontal distractor adjusted r = 0.65, all p < 0.001). A curvilinear line could be fitted to these data as displayed in Fig. 4.

With regards to the vertical time, 5% more time was needed on average to read the horizontal sheet and 13% more time to read the horizontal sheet with distractors. Specifically, there was a median delay of 3.2 s when the selective search was performed in the horizontal sheet compared to the vertical. When distractors are introduced, the median temporal increase was 9.2 s compared to the vertical. Likewise, the median difference in reading time between the horizontal sheets with and without distractors was 6.0 s. Quality of test performance showed an increased demand in horizontal sheets (without distractors 91.8%; with distractors 89.8%) compared to vertical quality index (98.8%).

Concerning the analysis and comparison of the results obtained with ADEMd and UFOV tests, the following mean values were obtained in the three subtests of the UFOV: 24.71 ± 23.69 ms (1 m), 73.15 ± 83.88 ms (2 m), and 129.18 ± 123.26 ms (3 m). Other results that were extracted from the analysis if the UFOV test outcomes were mean scores for risk (1.45 ± 0.73), processing (1.15 ± 0.46), divided attention (1.29 ± 0.49), and selective attention (1.04 ± 0.20). Table 3 summarizes the results of the correlation analysis among ADEMd and UFOV test outcomes.

**Table 3:** Spearman correlation coefficients among the adjusted times obtained with the ADEMd test and the results obtained with the UFOV test in a subsample of 75 subjects.

	UFOV							ADEMd		
	1	2	3	Risk	PV	DA	SA	Vaj	Haj	Hdaj
Vaj	0.10	0.17	0.17	0.23	0.20	0.19	0.13	1.00	0.90**	0.82**
Haj	0.06	0.15	0.27*	0.22	0.14	0.19	0.14	0.90**	1.00	0.88**
Hdaj	0.32**	0.32**	0.42**	0.31*	0.24	0.25*	0.06	0.82**	0.88**	1.00

*p < 0.05; **p < 0.01. *Vaj* vertical adjusted time, *Haj* horizontal adjusted time, *Hdaj* horizontal distractor adjusted time, *PV* processing velocity, *DA* divided attention, *SA* selective attention, *UFOV* useful field of view test, *ADEMd* Adult Developmental Eye Movement test with distractors.

## Discussion

The visual system utilizes different parameters during the visual search task for information. In order to evaluate these parameters, development eye movements and visual attention and facilitate their study at a clinical level, this investigation offers the possibility of characterizing them through a simple visual-verbal modified reading test. The ADEMd test allows the clinician to evaluate the eye movements and visual attentional behaviour in adults with no requirement of sophisticated equipment. As found in previous studies with the DEM^17^ test version for children^1^, vertical times in adults were lower (better) than the horizontal. Garzia et al.^1^ considered that the vertical response with the DEM test was mainly useful for the evaluation of the rapid automaticity naming (RAN; i.e., the automaticity of retrieval of the names of words or numerals^2^). On the other hand, the horizontal response was a combined measure of RAN and ocular motor (fixation and saccadic) capabilities^3^. Other authors have also reported in adults the presence of lower vertical times compared to the horizontal with the ADEM^10,11^. The aim of the current project was to validate the ADEMd test by performing intrasession repeatability, test–retest, and validity analyses.

The ADEMd test includes two-digit numbers, which implies an increase in the cognitive visual-verbal demand when the numbers are named fast compared to one-digit numbers^12^. This higher requirement altogether with what Larter et al.^2^ designated as spatial search factor (i.e., demand on the visual system to process information about the relative position and orientation of stimuli), increase the attentional and visual-verbal requirements of the test. Furthermore, the necessity of naming the numbers and not the letters increases the difficulty of the task in terms of overt and covert attention, and cognitive demands. Bearing in mind that similar areas of the brain use similar neurologic mechanisms for the attentional and oculomotor control^2^, these authors suggested that the DEM can be of good predictive value in the identification of a reduced saccadic function^2^. With the ADEMd test, the demand is even higher as an election must be done with divided attention into several stimuli that appear simultaneously in the field of vision. Therefore, we hypothesized that the addition of the distractors would be increasing the visual-verbal and attentional requirements of the test, and thus, it is acceptable that the time required to read the horizontal distractor sheet increases and the quality index decreases. These lower values of the quality performance index are justified by a higher probability of doing mistakes within a more demanding task.

The intrasession repeatability analysis performed in the current study revealed that the vertical and horizontal times measured with the test were repeatable and therefore consistent. An excellent correlation was found between both vertical adjusted times, and also between the adjusted time required to read the first 40 numbers of the horizontal sheet and that required for the other 40 numbers. Orlansky and colleagues^18^ found similar results but evaluating the reliability of the DEM test in children. Specifically, these authors found that the within-session repeatability for vertical and horizontal adjusted times were good to excellent. However, these authors concluded that clinicians should be cautious about using the DEM test in isolation for reaching a diagnosis as the repeatability for ratios and errors was more limited^18^. It should be considered that the DEM and also ADEMd are not only characterizing ocular movements but also visual attentional behaviour. Ayton et al.^3^ demonstrated in a study evaluating 158 children aged 8 to 11 years with the DEM test and also with an infrared eye tracker, that there was no significant correlation between any component of the DEM test performance and quantitative eye movement parameters (gain, latency, asymptotic peak velocity, and number of corrective saccades). Cohen et al.^19^ found in another study evaluating 66 children aged 8–10 years that the DEM score was correlated with asthenopic symptoms, but not with the results of a reading comprehension test. In contrast, Webber and colleagues^20^ reported that DEM outcomes could identify children whose Visagraph recorded eye movement patterns show slow reading rates. Therefore, the results of the DEM and ADEMd tests should not be used to perform consistent diagnosis of ocular movement alterations and should be used in combination with other clinical tests. Specifically, the DEM test has been stated to be not recommendable to diagnose eye movement difficulties in patients with poor reading ability. More research is needed in the diagnostic ability of the DEM test and the ADEMd test validated in the current study.

Besides the intrasession repeatability, a test–retest or intersession analysis was performed using the intraclass correlation coefficient and Bland–Altman method. The ICC ranged from 0.81 for horizontal distractor adjusted in subjects younger than 50 years, to 0.97 for vertical adjusted in subjects older than 50 years. This is consistent with the results of previous studies evaluating the test–retest repeatability of the DEM test in children^7^ and confirms good levels of intersession repeatability with the ADEMd test in adults. In any case, more variability and slightly poorer performance of the ADEMd test were observed in the subjects younger than 50 years, even though they had good performance levels. Rouse et al.^21^ tested 30 third grade children and retested two weeks after and found poorer inter-session repeatability. Possibly, the repeatability is more limited in younger patients due to relatively more difficulty in maintaining attention. It should be considered that the DEM and ADEMd test evaluates not only the oculomotor component but also the visual attentional behaviour. The Bland–Altman analysis in our study confirmed this trend of more consistent results in older patients, with lower limits of agreement.

Finally, the validity of the ADEMd test was evaluated in the current series. For such purpose, comparisons between different age ranges were carried out. It should be considered that there is no gold standard defined for the clinical analysis of ocular movements. A positive significant correlation was found between age and vertical and horizontal adjusted times. This means that the older the patient was, the more delayed answer was observed, with statistically significant differences in all adjusted times in the different age subgroups. While caution should be applied since we did not directly measure eye movement function, these increases in the adjusted times may be related to a decrease in the efficiency of central visual processing with ageing^22^, as well as to the deterioration of saccadic eye movements^23^ and the worsening of tracking ocular movements in elderly patients compared to young^11,24,25^. Also, these worst results for the older patients could be related to detriments in the overt or covert attention^14^, which deficit may not imply a deficit in the oculomotor system^13^. The ADEMd test was also able to detect variability between vertical and horizontal adjusted times with age. Likewise, there was more significant variability in the results of the oldest patients besides the increase in the magnitude of the adjusted times. This is consistent with studies showing a higher level of interindividual variability in older patients due to differences in the evolution of the ageing process in each individual case^24^. The significant increase in the adjusted time of the horizontal distractor sheet according to age is consistent with the results of previous studies showing that the response in elderly people is slower and less precise in disorganized scenes which require longer fixations under divided attention conditions^26^. Similarly, as happens with overt attention, it has been confirmed that velocity in the attentional selection from one task to another tends to be reduced with age^27^.

For the evaluation of the validity of the test, a comparison of the behaviour of the ADEMd test and the attentional field UFOV (Useful Field of View) test was performed considering that the ADEMd test is also evaluating an attentional visual factor. The UFOV test has been shown to be useful and reliable to evaluate the useful field of view in healthy subjects as well as in other ocular conditions^28,29^. In our sample, processing velocity (subtest 1) and divided attention problems (subtest 2) measured with the UFOV test showed a poor but statistically significant positive correlation with horizontal adjusted. Likewise, the difficulty in the selective attention (subtest 3) showed a moderate positive correlation with horizontal distractor adjusted and a poor but statistically significant correlation with horizontal adjusted. Therefore, the UFOV indices of divided and selective attention were correlated mainly with the adjusted time required to read the horizontal distractor sheet, which is the sheet with more requirements in terms of visual attention. The limited level of correlation among UFOV indices and horizontal distractor adjusted may be partially due to the necessity of including the impact of the ocular movement factor. Several studies have confirmed the interconnection between visual attention and ocular movements. Indeed, attention has been shown to have a crucial role in reading speed, in the ocular movements occurring during reading and in the generation of voluntary saccadic movements^30,31^.

Although a considerably large sample size was used and all the procedures were carefully designed and supervised, there are some limitations and future research lines that should be listed. First, it is worth highlighting that this test is not directly measuring the oculomotor function, future studies should support this hypothesis with the use of an eye-tracker device. Also, further research should investigate the relationship between the adjusted times and common visual measures in the literature (e.g., saccadic latencies, smooth pursuit, fixation, visual search reaction time), in which other tests of cognitive and visual processing performance could have been included to compare the results and further validate the ADEMd test. It should be mentioned that previous expert literature has found a learning effect in children within the use of the DEM^18,32,33^, while, on the other hand, no studies have assessed this in adults with the ADEM or ADEMd. The mean difference between the test and retest in the present study was 0.33 s (group younger than 50) and 2.4 (group older than 50) for the vertical adjusted time, 1.86 (group younger than 50) and 6.09 (group older than 50) for the horizontal adjusted, and 0.52 (group younger than 50) and 5.07 (group older than 50) for the horizontal with distractors adjusted, being these differences non-significant (except for the horizontal sheet in the group older than 50 years). Finally, it could be interesting to correlate the performance in the ADEMd with visual difficulties in daily activities such as driving.

In conclusion, the ADEMd test may be a useful clinical tool to evaluate the combined interaction of ocular movements and visual attention behaviour. This test can provide consistent repeated measurements as well as to detect the variations in the response associated with age. Perhaps the modification of the ADEM with the addition of distractors increases the visual processing requirements of the test. This study is just one step in the process of validating the new ADEMd. Further research should be conducted to report if this modification might be useful in the identification and evaluation of individuals with neurodegenerative illnesses, such as multiple sclerosis (a nerve transmission problem), Alzheimer disease (a cognitive problem), or individuals with a history of traumatic brain injury. Likewise, other applications of this test for situations and areas in which high ocular movement and visual attention demands are required, such as driving, should be investigated.

## Methods

### Subjects

All subjects were informed about their inclusion in the study and gave informed consent to participate in accordance with the tenets of the Declaration of Helsinki (as revised in 2013). The Ethics Committee of the University of Valencia approved this study. Measurements were performed in a private optometric clinic (Valencia, Spain).

Inclusion criteria were: (1) not having previous experience in similar tests, (2) corrected distance visual acuity of 0.2 logMAR or better, (3) spherical equivalent refraction between − 6.00 and + 6.00 dioptres, and (4) absence of oculomotor alterations. Exclusion criteria were any active ocular or systemic disease, cataract, abnormal retinal function, and psychiatric problems.

The study was conducted in a sample of 302 subjects. The age ranging from 20 to 86 years (mean age: 52.2 ± 18.7 years). Table 4 presents further sociodemographic characteristics of the sample and the Subsamples 1 and 2 used for reliability and validity, respectively (see “Reliability analysis” and “Validity analysis” sections).

**Table 4:** Sociodemographic characteristics of the sample and each of the two subgroups.

	Total sample (n = 302)	Subsample 1 (n = 40)	Subsample 2 (n = 75)
Age (years)	52.18 ± 18.73	53.28 ± 19.12	50.27 ± 15.76
**Age groups**			
20.0–24.9	26 (8.6%)	8 (20.0%)	14 (18.7%)
25.0–34.9	55 (18.2%)	4 (10.0%)	8 (10.6%)
35.0–44.9	31 (10.3%)	5 (12.5%)	8 (10.6%)
45.0–54.9	32 (10.6%)	5 (12.5%)	20 (26.6%)
55.0–64.9	65 (21.5%)	8 (20.0%)	16 (21.4%)
65.0–74.9	61 (20.2%)	7 (17.5%)	5 (6.7%)
> 75.0	32 (10.6%)	3 (7.5%)	4 (5.3%)
**Sex**			
Female	159 (52.6%)	29 (72.5%)	37 (49.3%)
Male	143 (47.4%)	11 (27.5%)	38 (50.7%)
**Academic level**			
Basic	112 (37.1%)	13 (32.5%)	26 (34.7%)
Middle	81 (26.8%)	10 (25.0%)	15 (20.0%)
Higher	109 (36.1%)	17 (42.5%)	34 (45.3%)

Values are presented as mean ± standard deviation or frequencies and percentages between parenthesis.

### Modification of the ADEM test (ADEMd)

**Figure 5:**
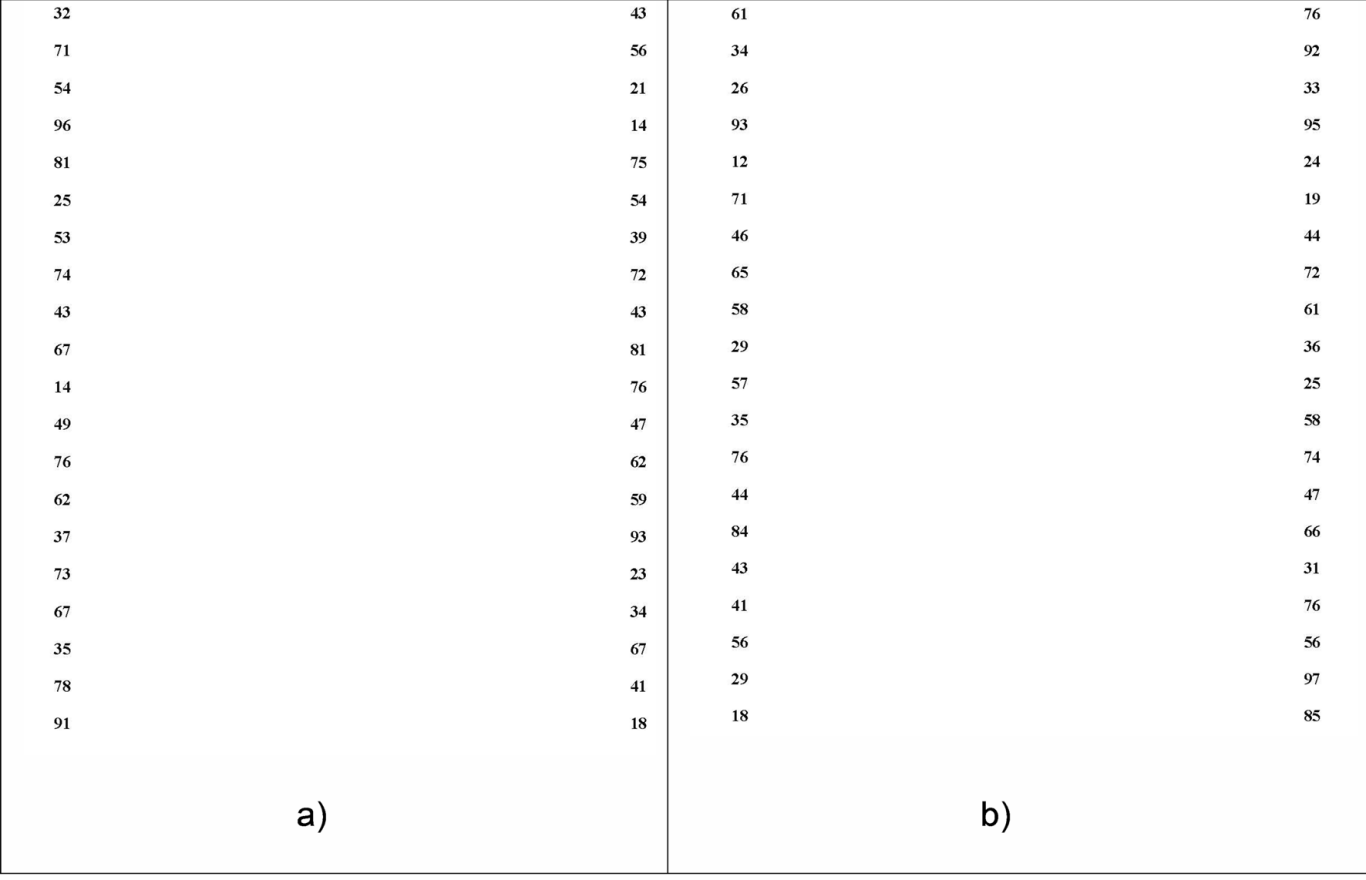
Vertical sheets (V1 and V2) of the ADEMd test.

**Figure 6:**
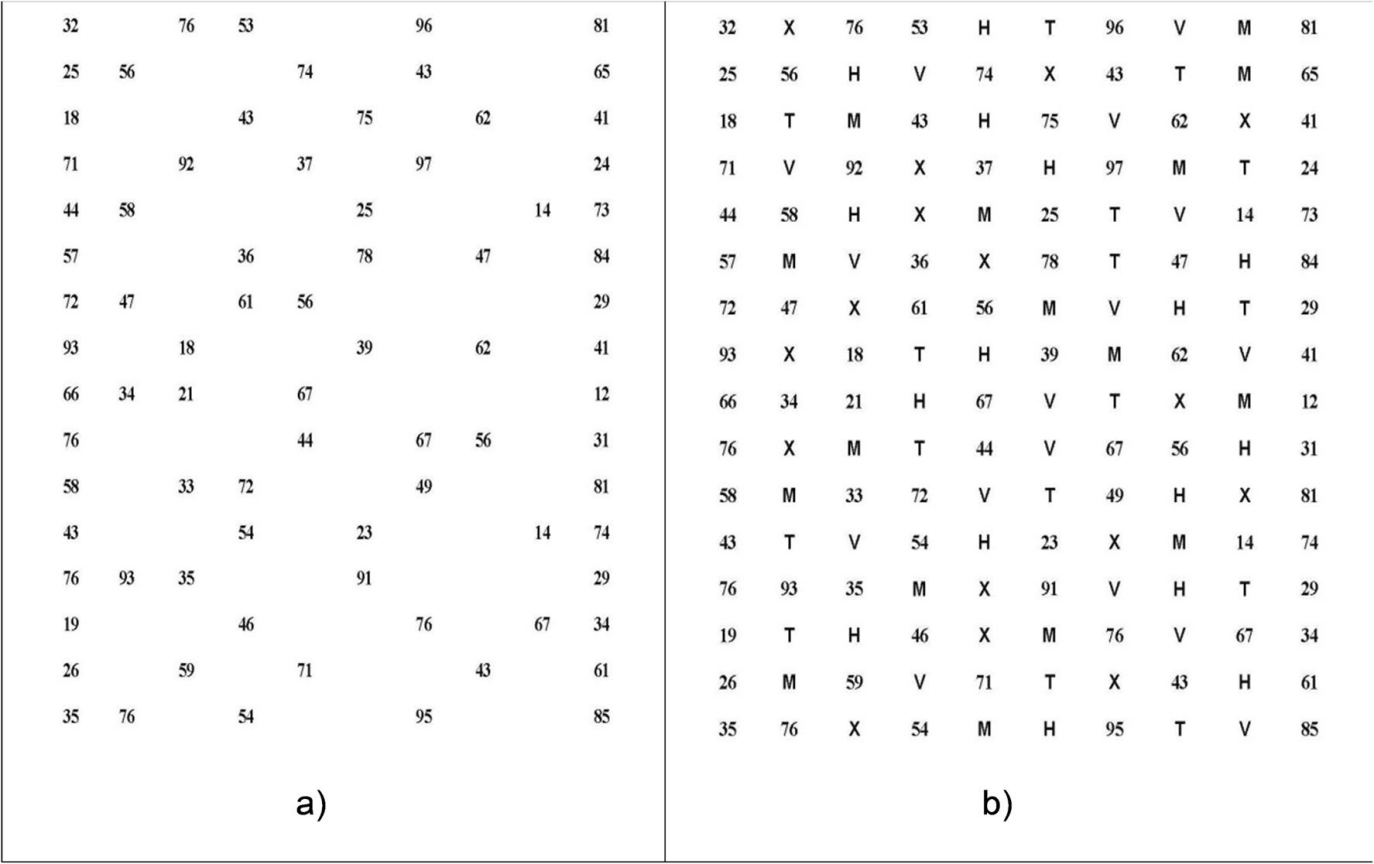
Horizontal (H) and horizontal with distractors (Hd) sheets of the ADEMd test.

The main goals of the modification of ADEM were to overcome the limitations of the original version in terms of confusion of numbers and to increase the demands in visual processing. The ADEMd test has been designed to avoid the possible effect of confusion of using 80 different numbers between the vertical and horizontal sheets that happened with the original ADEM test, as was observed in the pilot study of its validation^10^. For overcoming this limitation, the test has been partially modified by including a new horizontal sheet with the same numbers used in the vertical sheets, as suggested by Powell et al.^11^. Likewise, a new horizontal sheet has been added with the aim of increasing the difficulty of cognitive processing. This sheet includes distractors (letters) between the numbers introducing a more demanding factor of spatial search. Specifically, the horizontal distractor sheet includes five different types of letters (H, M, T, V and X) between the numbers, allowing not only the evaluation of oculomotor alterations but also the patient’s attention ability. The numbers on this horizontal distractor sheet are the same as those included on the first horizontal sheet. Therefore, the ADEMd test is composed of four different sheets, two vertical (Fig. 5), and two horizontal (Fig. 6).

Both vertical sheets require an organized task of low spatial demand. Numbers are distributed in two columns that are widely separated (20 numbers each). The subject has to move the eyes vertically in each column while naming the numbers from top to down. This task requires minimal fixating saccadic movements and can be considered an operational cognitive task. Thus, selective attention on an only information source and a unique task (identification and naming of numbers) is needed. The first horizontal sheet is more demanding as 80 numbers are presented in 16 horizontal lines, with a space between them randomly distributed. Numbers should be read from left to right and from top to down. It requires a planification system and a selective search with ocular movements that are mainly horizontal, with fixations and saccades. In the horizontal distractor sheet, there is a demand for precise horizontal ocular movements with attention divided into identifying and naming numbers and identifying but not naming letters.

The ADEMd test uses Times New Roman letters of size 11 that is equivalent to a Snellen resolution of 20/80 when presented at a distance of 40 cm. The extremes of the numbers of the columns of the vertical sheets subtend an angle of 17.4° vertically and 17.3° horizontally when viewed at 45 cm. The vertical angular separation between numbers is 1.2°, which is an area that falls within the central retina. The extremes of the numbers of the lines of the horizontal sheets subtend an angle of 17.4° vertically and 17.1° horizontally when viewed at 45 cm. The horizontal angular separation between characters varies from 1.91° to 9.78°, with a mean value of 4.39 ± 1.93°.

### Measurement procedure with the ADEMd test

The subject was first asked to read out loud the numbers of the first vertical sheet as fast as possible. Meanwhile, the examiner was recording the speech with a conventional recorder. Afterwards, the same procedure was performed with the second vertical sheet and the first horizontal sheet. At this point, the subject was asked to read only the numbers of the horizontal distractor sheet. The subject had to continue reading when a mistake was done, maintaining the reading rhythm as fast as possible. Once finished, the recorded speech was analysed to define the time needed to read each sheet and the mistakes made; mistakes consisted of additions or omissions of numbers, and any letter read.

The scoring of the test was calculated considering the following guidelines:

1. Adjusted vertical time, which is a measure of the naming speed or automaticity:
  $$ {\text{Vertical}}\;{\text{adjusted}}\;{\text{time}}\;({\text{s}}) \, = {\text{ Vertical}}\;{\text{time }} \times \, 80{/}(80 \, - {\text{ omissions }} + {\text{ additions}}) $$
  where vertical time are the seconds needed to read both vertical sheets. It takes into account the number of omissions and additions of numbers.
2. Adjusted horizontal times, which is an indirect evaluation of fixations and saccades combined with the automaticity in naming numbers and divided attention:
  $$ \begin{gathered} {\text{Horizontal}}\;{\text{adjusted}}\;{\text{time}}\;{\text{(s) }} = {\text{ Horizontal}}\;{\text{time }} \times \, 80{/}\left( {80 \, - {\text{ omissions }} + {\text{ additions}}} \right) \hfill \\ {\text{Horizontal}}\;{\text{distractor}}\;{\text{adjusted}}\;{\text{time}}\;({\text{s}}) \, = {\text{ Horizontal}}\;{\text{distractor}}\;{\text{time }} \times \, 80/\left( {80 \, - {\text{ omissions }} + {\text{ additions }} + {\text{ letters}}} \right) \hfill \\ \end{gathered} $$
  where horizontal and horizontal distractor times are the seconds needed to read each horizontal sheet. It takes into account the number of omissions and additions of numbers, and the number of letters read.
3. Calculation of three different ratios: Horizontal adjusted/Vertical adjusted, which compares horizontal (oculomotor control with automaticity) and vertical (automaticity) levels; Horizontal distractor adjusted/Vertical adjusted, which compares the attentional horizontal level (oculomotor control with automaticity incorporating the distractor task) with the vertical level (automaticity); and Horizontal distractor adjusted/Horizontal adjusted, which compares attentional horizontal (more demanding task due to the presence of distractor elements) with the horizontal (oculomotor control) levels.
4. Quality of test performance:
  $$ \begin{gathered} {\text{Quality}}\;{\text{Vertical}}\;{\text{Test}}\;{\text{Performance }} = \, 100 \, {-} \, \left( {\left( {{\text{total}}\;{\text{number}}\;{\text{of}}\;{\text{vertical}}\;{\text{errors/}}80} \right) \, \times \, 100} \right) \hfill \\ {\text{Quality}}\;{\text{Horizontal}}\;{\text{Test}}\;{\text{Performance }} = \, 100 \, {-} \, \left( {\left( {{\text{total}}\;{\text{number}}\;{\text{of}}\;{\text{horizontal}}\;{\text{errors/}}80} \right) \, \times \, 100} \right) \hfill \\ {\text{Quality}}\;{\text{Horizontal}}\;{\text{distractor}}\;{\text{Test}}\;{\text{Performance }} = \, 100 \, {-} \, \left( {\left( {{\text{total}}\;{\text{number}}\;{\text{of}}\;{\text{ horizontal}}\;{\text{distractor}}\;{\text{errors/}}80} \right) \, \times \, 100} \right) \hfill \\ \end{gathered} $$
  The higher the ratio is, the higher is the quality of test performance.

### Reliability analysis

The intrasession repeatability was evaluated first. For such purpose and while the vertical sheets had the same structure, the correlation (Spearman–Brown correlation coefficient) between the adjusted time required to read the first and the second vertical sheet was considered as a measure of the internal consistency of the test. Similarly, the correlation (Spearman–Brown correlation coefficient) between the time required to read the first and the second half of the horizontal and horizontal distractor sheets was analysed.

Besides the internal consistency of the test, a test–retest analysis was also performed (intersession repeatability). This analysis was only done in a random subsample of 40 subjects from the total sample (of which, 29 were also involved in the validity analyses). This subsample was divided into two subgroups according to the age: 20 subjects with an age of 50 years or more (mean age: 70.7 ± 8.3 years), and 20 subjects younger than 50 years (mean age: 32.8 ± 8.5 years). The test was repeated after 12 ± 3 days since the first evaluation. The intraclass correlation coefficient (ICC) and Bland–Altman method were used for the analysis of the outcomes obtained.

### Observational ability analysis

This analysis refers to the patient’s ability to show the same behaviour and the ability of the examiner of grading exactly the same proceeding. This was analysed by evaluating the intrasession repeatability and the results of test–retest experience, as previously described.

### Validity analysis

As there are no references or gold standards for the evaluation of saccadic eye movements, the analysis of the validity of the test was performed by comparing the results within different age ranges. It is well-known that there is a deterioration of saccadic eye movements with aging^23^, and this should be detected with the ADEMd test. Specifically, the correlation (Spearman correlation coefficient) among age and adjusted times was investigated. Likewise, the behaviour of the ADEMd test and the attentional UFOV (Useful Field of View) test^28^ were compared, and the correlation between them was investigated (Spearman correlation coefficient). This last analysis was done in 75 healthy subjects with a mean age of 50.3 ± 15.8 years. These subjects were a subsample of the ADEMd subjects. They were chosen at random.
